# Platelet to Lymphocyte Ratio Associated with Prolonged Hospital Length of Stay Postpeptic Ulcer Perforation Repair: An Observational Descriptive Analysis

**DOI:** 10.1155/2021/6680414

**Published:** 2021-03-09

**Authors:** Omer Al-Yahri, Tamer Saafan, Husham Abdelrahman, Ammar Aleter, Ali Toffaha, Mustafa Hajjar, Hesham Aljohary, Rashad Alfkey, Ahmad Zarour, Saif Al-Mudares, Ayman El-Menyar

**Affiliations:** ^1^Department of Surgery, Acute Care Surgery, Hamad Medical Corporation, Doha, Qatar; ^2^Department of Surgery, General Surgery, Hamad Medical Corporation, Doha, Qatar; ^3^Department of Surgery, Trauma Surgery, Hamad Medical Corporation, Doha, Qatar; ^4^Clinical Medicine, Weill Cornell Medical College, Doha, Qatar

## Abstract

**Background:**

The predictive role of platelet to lymphocyte ratio (P/LR) in patients with perforated peptic ulcer (PPU) is not well-studied. We aimed to investigate the association between the P/LR ratio and the hospital length of stay (HLOS) for surgically treated PPU.

**Method:**

This is a retrospective observational study for surgically treated adult cases of PPU at Hamad Medical Corporation during the period from January 2012 to August 2017. Patients were categorized into two groups based on their HLOS (<I week vs. >I week). The receiver operating characteristic (ROC) curve was plotted to determine the cutoff value for lymphocyte count, neutrophil to lymphocyte ratio, and P/LR ratio for predicting the prolonged hospitalization.

**Results:**

One hundred and fifty-two patients were included in the study. The majority were young males. The mean age was 38.3 ± 12.7 years. Perforated duodenal ulcer (139 patients) exceeded perforated gastric ulcer (13 patients). The HLOS > 1 week was observed in 14.5% of cases. Older age (*p* = 0.01), higher preoperative WBC (*p* = 0.03), lower lymphocyte count (*p* = 0.01), and higher P/LR ratio (*p* = 0.005) were evident in the HLOS > 1 week group. The optimal cutoff value of P/LR was 311.2 with AUC 0.702 and negative predictive value of 93% for the prediction of prolonged hospitalization. Two patients died with a mean P/LR ratio of 640.8 ± 135.5 vs. 336.6 ± 258.9 in the survivors.

**Conclusion:**

High preoperative P/LR value predicts prolonged HLOS in patients with repaired perforated peptic ulcer. Further larger multicenter studies are needed to support the study findings.

## 1. Introduction

Peptic ulcer disease (PUD) is a common medical and surgical condition. It affects 4 million people a year worldwide [1, 2]. Treatment is medical; however, most of the complications are treated surgically. Peptic ulcer perforation (PPU) is a significant complication that represents around 5% of all abdominal surgical emergencies and complicates 5% of patients with PUD. Peptic ulcer perforation is the second most common complication after bleeding [2, 3].

Laparoscopy offers advantages over open surgery and does reduce the length of hospital stay in PPU; however, the length of hospital stays varies widely. The mortality rate is high that can reach 30% [3].

The literature has multiple proposed clinical predictors of outcomes and prognostic scoring systems due to the associated high morbidity and mortalities. Old age, late presentation, low admission blood pressure, and comorbidities are examples of these predictors. There are many scoring systems built to predict morbidity and mortality in the PPU; however, these scoring systems are complex, difficult to measure, not easy to calculate, and in real-life, usually not routinely utilized [4–6].

The length of hospital stay is an important clinical outcome after surrey as it is a measure of the quality of care. It increases in patients with high morbidity and inversely shortened in patients with excellent outcomes [7–9].

Laboratory markers' use for prognostication of surgical procedures is a new and hot area in medical research. They have currently been investigated on how to help in diagnosis and in predicting the progression and outcome of various diseases. Several biomarkers were found to reflect the severity of the underlying inflammatory diseases, the host reactions, immune response, and the prothrombotic status of patients [10–14]. The platelet to lymphocyte ratio (P/LR) is an interesting example of these markers. It has been studied in several chronic inflammatory diseases and had a significant predictor of outcomes. [15–18] There is an increasing evidence that simple inflammatory biomarkers as P/LR as well as neutrophil to lymphocyte ratio (N/LR) can act as reliable predictors of a variety of medical conditions such as brain infarct, cerebral hemorrhage, and acute coronary syndrome [19–21]. The higher preprocedural P/LR predicts the short- and long-term prognosis after percutaneous revascularization interventions [19–21].

Furthermore, the N/LR aids in the diagnosis of PPU and found to be elevated in the ICU critically ill patients. Moreover, it could predict mortality in patients with complex surgery of the upper gastrointestinal tract [22]. The P/LR, in surgical patients, has been studied in acute conditions like mesenteric ischemia, PPU, and trauma [13–17]. For example, in patients with mesenteric ischemia, a high P/LR reliably predicts a bad prognosis with a high 30-day mortality rate. Also, P/LR was found to be high in nonsurvival traumatized patients without sepsis [13, 15, 16]. The P/LR was reported as a predictor of high mortality in patients who had surgery for PPU in a study by Aydin and Pehlivanlı [23]. The latter is the only study utilizing P/LR in PPU; however, the sample size was small (only 23 patients), and not validated yet. We aimed to investigate the association between the P/LR ratio and one of the primary PPU outcomes which is the longevity of hospital stays. We hypothesized that a high P/LR ratio is associated with prolonged hospitalization post-PPU repair.

## 2. Methods

This is a retrospective observational cohort study for surgically treated adult cases of PPU in Hamad Medical Corporation (HMC) during the period between January 2012 and August 2017. The inclusion criteria were all consecutive adult patients (>14 years old), admitted with PPU to HMC, and were surgically treated with complete preoperative laboratory records.

Exclusion criteria included patients aged below 14 years, PPU was due to nonpeptic ulcer cause, such as tumors, trauma, or iatrogenic, and relevant if preoperative laboratory records were not available.

Data (clinical and laboratory) were collected for the patients operated for PPU from the general and acute care surgery departments at Hamad Medical Corporation. Specifically, we collected data on age, gender, initial labs (hemoglobin level, platelet count, leukocyte count, absolute neutrophil count, lymphocyte count, N/LR, P/LR, and mean platelet volume), operative reports (localization of perforation), and clinical outcomes (hospital length of stay and mortality). We calculated the P/LR and N/LR for all the patients.

All patients underwent initial management in the emergency department with IV fluids and antibiotics and subsequently underwent surgical treatment (laparoscopic, open, or laparoscopic converted to open approaches) and proceeded according to the intraoperative findings. The standard operative procedure was simple repair using Vicryl sutures and omental patch and wash out, and in case of deformed sclerosed duodenum or huge or neglected cases, diversion with gastroduodenostomy was done.

The postoperative care is typically carried on in the surgical ward or the critical care unit. Gradual resumption of the oral intake starts on the following morning and advanced slowly as tolerated. Patients received an IV antibiotic regimen usually cefuroxime plus metronidazole along with IV ranitidine or proton pump inhibitor, and in case of duodenal perforation where H. pylori infection is considered, triple therapy was added as soon as the patient recovered for 4-6 weeks.

Patients were categorized into 2 groups based on the HLOS (<1week vs. >one week). This 1-week cutoff value was extrapolated from the average HLOS in prior studies [24, 25].

Approval for this retrospective study was obtained from the Institutional Review Board (IRB) and Medical Research Center (MRC) with reference number MRC/0058/2018 and research proposal number 17168/17.

### 2.1. Statistical Analysis

Data were presented as proportions, medians (minimum–maximum range), or mean (±standard deviation; SD) as appropriate. Study variables were analyzed and compared according to hospital length of stay (HLOS ≤ 1 week versus >1 week). Differences between categorical variables were analyzed using the chi-square or Fisher's exact test, whereas Student's *t* test was performed to compare continuous variables, whenever applicable. The receiver operating characteristic (ROC) curve was plotted to determine the cutoff value for lymphocyte count, N/LR, and P/LR for predicting the prolonged hospitalization (>1 week). The area under the curve (AUC) was used to compare the discriminatory power of the lymphocyte count, N/LR, and P/LR, with an AUC of 1.0 considered as perfect discrimination and 0.5 considered as equal to chance. The sensitivity, specificity, positive predictive value (PPV), negative predictive value (NPV), positive likelihood ratio, negative likelihood ratio and accuracy of the lymphocyte count, N/LR, and P/LR in predicting the prolonged hospitalization were determined. A two-tailed *p* value < 0.05 was considered significant. Data analysis was carried out using IBM SPSS Statistics for Windows, Version 21.0., Armonk, NY, USA.

## 3. Results

During the study period, a total of 152 patients met the inclusion criteria and were included in the study. There were 151 males (99.3) and only one female (0.7%); the mean age of the cohort was 38.3 ± 12.7 years (Table 1). All patients underwent surgical treatment. Perforations were in the duodenum in 139 patients (91.4) and in the stomach in 13 patients (8.6%) based on the operative report. The mean length of hospital stay was 8.35 ± 21.5 days. Two patients (1.3%) died during the first three months of surgery.

Table 2 shows a comparison between the groups of the study based on the HLOS. There was a statistically significant difference between the two groups regarding age; the mean age was higher in the HLOS > 1 week's group (49.9 ± 3.31 years) compared with the HLOS < 1 week's group (36.3 ± 0.97 years: *p* = 0.01). The leukocyte count was significantly higher in the HLOS > 1 week's group (15.7 ± 4.2) compared to the HLOS < 1 week's group (14.2 ± 0.9; *p* = 0.03). The neutrophil count was higher in the HLOS > 1 week's group (11.2 ± 0.40) compared to the HLOS < 1 week's group (10.6 ± 1.9; *p* = 0.05), but it did not reach statistical significance. The lymphocyte count was significantly higher in the HLOS < 1 week's group (1.2 ± 0.08) compared to the HLOS > 1 week's group (0.8 ± 0.11; *p* = 0.01). The P/LR was higher in the HLOS > 1 week's group (497.6 ± 70.1) compared to the HLOS < 1 week (318.6 ± 23.3) with a *p* value of 0.005. The N/LR was comparable between the 2 groups.

The two deaths in the cohort (1.3%) were found in the prolonged HLOS group. The mean P/LR ratio was higher in the deceased (640.8 ± 135.5) compared to 336.6 ± 258.9 in the survivors (Table 3). In comparison to survivors, patients who died were more likely to be older in age (69.5 ± 14.8 vs. 37.9 ± 12.3), had significantly higher platelet count (440.5 ± 314.6 vs. 264.9 ± 85.9), and prolonged hospitalization (49.5 ± 26.2 vs. 8.0 ± 21.9; *p* < 0.05).

**Figure 1:**
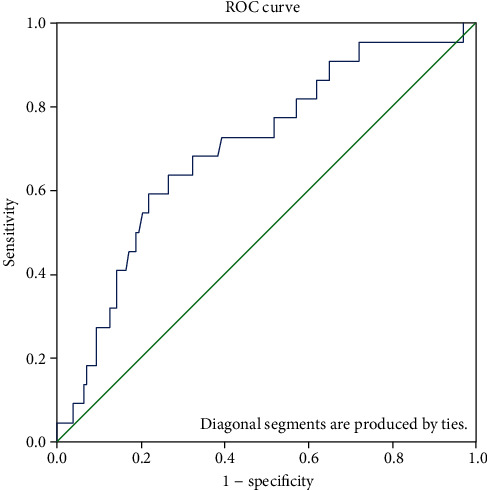
Receiver operating characteristic (ROC) curve graphs showing the sensitivity and specificity of the platelet to lymphocyte ratio values in the determination of the hospital length of stay > 1 week.

Table 4 and Figure 1 demonstrate the discriminatory power for lymphocyte count, neutrophil to lymphocyte ratio, and platelet to lymphocyte ratio for the prediction of prolonged hospital stay (>1 week).

The ROC curve showed an area under the curve (AUC) for the prediction of prolonged hospitalization based on P/LR to be 0.702. The optimal cutoff value of P/LR was 311.2 for the prolonged hospitalization with sensitivity (68.2% (45.1-86.1)) and specificity (68% (59.2-75.9)). It had also a higher negative predictive value and accuracy of 92.6% (87-95.9) and 68% (59.9-75.4), respectively. On the other hand, the ROC curve analysis, for lymphocyte count and N/LR, showed poor diagnostic value with lesser AUC, i.e., 0.331 and 0.499, respectively. In addition, both the laboratory tests have lower sensitivity, specificity, positive predictive value, and accuracy for the prediction of prolonged hospitalization.

## 4. Discussion

This is a unique study that reports the utility of a simple mediator of inflammation such as P/LR for predicting the length of hospital stay in surgically treated PPU. The study reveals that the prognostic role of P/LR outperforms the N/LR in patients who required prolonged hospital course. The majority of cases had a perforated duodenal ulcer, while gastric perforation happened in 8.6%. Localization of peptic ulcer perforation is known to be more in the duodenum than the stomach [26, 27]. Advanced age was significantly associated with long HLOS in the analysis, the mean age of patients with HLOS ≤ 7 days was 36.3 ± 0.97 while it was 49.9 ± 3.3 years in HLOS > 7 days. This could be justified by the higher distribution of comorbidities in patients > 50 years old. Gasparyan et al. mentioned a closer prediction of advanced age (≥65 years) to be associated with a longer HLOS [10]. Sivaram et al. also reported a similar result as age more than 50 years was associated with a longer HLOS [7].

The average length of hospital stay among our patients was 8.35 days. However, the majority of patients (85.5%) stayed less than one week in the hospital. The average HLOS reported in the literature is between 7 and 11 days [7, 24, 25, 28].

The HLOS generally reflects the disease course in the hospital and its outcome. The longer HLOS reveal a nonstraightforward hospital course and worse outcome in terms of morbidity and mortality. The prolonged HLOS is well known to have drawbacks on the cost and time spent by patients and treating facilities [3, 29].

Knowing the importance of HLOS derived us to explore the predictors of HLOS with special attention to the biomarkers. This study reports a positive correlation between age, preoperative high P/LR, low lymphocyte count, and HLOS. Furthermore, the two deaths happened in the prolonged HLOS group who showed a high P/LR. No significant differences were found between the study groups in terms of the platelet count, neutrophil count, mean platelet volume, and N/LR.

The low lymphocyte count in the HLOS > 7-day group probably indicates slow cellular immunity response in the advanced age and comorbidities. The lymphocyte count is the key in determining the P/LR. The older age, high platelet count, high N/LR, P/LR ratios, and low lymphocyte count were associated with mortality in our study, which is consistent with Aydin and Pehlivanlı's study [23]. The discriminatory power of P/LR in the present study is reflected by the AUC 0.702 and NPV of 93%. So, the high P/LR ratio can be used as a marker to predict poor outcomes, namely, prolonged HLOS.

We reported only two in-hospital deaths (1.3%). The age may have contributed to the risk of death due to the limited physiologic reserve, less responsive immune system, high incidence of comorbidities, and malnutrition. The low mortality rate is partly due to the young age of our patients besides the health care system with easy access, free emergency care, and availability of immediate advanced surgical and critical care. Aydin and Pehlivanlı, though studied small sample size, reported a 17% death rate and a mean age of 54.5 years [23]. In patients who underwent coronary artery bypass surgery (CABG), the preoperative P/LR was found to be an independent risk factor for the development of arrhythmia and neurologic events, reoperation for sternum dehiscence, prolonged hospital length of stay, and mortality in the early postoperative period [30].

The link between the higher preoperative P/LR and postoperative complications remains unclear apart from the exaggerated inflammatory and altered immunity status. P/LR could reflect the balance between the body response to inflammation and immunity mediators. Platelets, as a contributor in the inflammatory response, and platelet-associated chemokines such as platelet factor 4 and connective tissue-activating peptide III can modulate inflammation; however, low lymphocyte counts may lead to inadequate immune responses [31].

## 5. Limitations

Our study has several limitations. It is a retrospective observational study, and thus, it may be subjected to all known limitations of retrospective studies. Selection bias and power of the study can not be ignored. Comorbidities were not recorded in detail, and their potential impact on outcome could not be evaluated. We believe that prospective and multicenter studies are needed to define further the role of these biomarkers in PPU and similar surgical emergencies.

## 6. Conclusion

High preoperative P/LR value predicts the length of hospital stay post-PPU repair. Multicenter contributions would provide a large sample and help to develop a piece of solid evidence for the utility of these prognostication markers to inform physician's critical decision makings and guide the intensity of care.

## Figures and Tables

**Table 1:** Demographics, laboratory results, and outcome of surgically treated perforated peptic ulcer patients (*N* = 152).

Age	38.3 ± 12.7
Gender	
Females	1 (0.7%)
Males	151 (99.3%)
Localization	
Stomach	13 (8.6%)
Duodenum	139 (91.4%)
Laboratory results	
Hemoglobin level (g/dL)	15.3 ± 2.8
Leukocyte count (×10^3^/mcL)	14.3 ± 10.9
Platelet count (×10^3^/mcL)	267.5 ± 92.1
Absolute neutrophil count (×10^3^/mcL)	11.01 ± 5.6
Lymphocyte count (×10^3^/mcL)	1.15 ± 0.8
Neutrophil to lymphocyte ratio	14.1 ± 15.3
Platelet to lymphocyte ratio	340.6 ± 259.8
Mean platelet volume	12.82 ± 50.7
Length of stay in hospital	8.35 ± 21.5
Outcome	
Discharged	150 (98.7%)
Death	2 (1.3%)

**Table 2:** Comparisons of the variables according to hospital length of stay (HLOS).

Variables	HLOS < 1 week (*n* = 130)	HLOS > 1 week (*n* = 22)	*p*
Age in years	36.3 ± 0.97	49.9 ± 3.31	0.01^ϯ^
	min: 19 max: 87	min: 24 max: 80	
Gender			
Male	129 (99.2%)	0 (0%)	0.7^×^
Female	1 (0.8%)	2 (100%)	
Localization			
Stomach	118 (90.8%)	21 (95.5%)	0.7^×^
Duodenum	12 (9.2%)	1 (4.5%)	
Laboratory results			
Hemoglobin level (g/dL)	15.38 ± 0.22	14.4 ± 1.03	0.4^∗^
	min: 7.2 max: 32.5	min:7.2 max: 30.9	
Leukocyte count (×10^3^/mcL)	14.2 ± 0.9	15.7 ± 4.2	0.03^∗^
	min: 1.8 max: 98.2	min: 2.0 max: 89.6	
Platelet count (×10^3^/mcL)	260.23 ± 7.9	307.71 ± 25.8	0.08^∗^
	min: 103 max: 610	min: 146 max: 663	
Neutrophil count (×10^3^/mcL)	11.2 ± 0.4	10.6 ± 1.9	0.05^∗^
	min: 0.4 max: 24.9	min: 1.4 max: 40.9	
Lymphocyte count (×10^3^/mcL)	1.2 ± 0.08	0.8 ± 0.11	0.01^∗^
	min: 0.2 max: 5.1	min: 0.2 max: 2.4	
Neutrophil to lymphocyte ratio	13.4 ± 1.2	19.3 ± 6.01	0.12^∗^
	min: 1.0 max: 101	min: 2.5 max: 128	
Platelet to lymphocyte ratio	318.6 ± 23.3	497.6 ± 70.1	0.005^∗^
	min: 56.6 max: 1316.7	min: 83.8 max: 1555.0	
Mean platelet volume	13.6 ± 5.2	9.2 ± 0.32	0.7^∗^
	min: 3.5 max: 606.0	min: 6.8 max: 12.0	
Outcome			0.001^×^
Alive	130 (100%)	20 (90.9%)	
Dead	0 (0%)	2 (9.1%)	

^∗^Mann-Whitney *U* test; ^ϯ^*t*-test; ^×^chi-square test.

**Table 3:** Characteristics of survivor and deceased.

Variables	Survivors (*n* = 150)	Deceased (*n* = 2)
Age in years	37.9 ± 12.3	69.5 ± 14.8
Gender		
Male	149 (99.3%)	2 (100%)
Female	1 (0.7%)	0 (0%)
Localization		
Stomach	13 (8.7)	0 (0%)
Duodenum	137 (91.3%)	2 (100%)
Hemoglobin level (g/dL)	15.32 ± 2.81	13.7 ± 0.63
Leukocyte count (×10^3^/mcL)	14.4 ± 11.4	17.1 ± 0.8
Platelet count (×10^3^/mcL)	264.9 ± 85.9	440.5 ± 314.6
Neutrophil count (×10^3^/mcL)	11.0 ± 5.6	15.6 ± 0.9
Lymphocyte count (×10^3^/mcL)	1.2 ± 0.8	0.7 ± 0.4
Neutrophil to lymphocyte ratio	13.9 ± 15.2	28.5 ± 16.9
Platelet to lymphocyte ratio	336.6 ± 258.9	640.8 ± 135.5
Mean platelet volume	12.9 ± 51.4	10.2 ± 0.8

**Table 4:** Discriminatory power analysis of preoperative laboratory results for the determination of prolonged hospital length of stay.

Variables	AUC	*p* value	Cutoff value	Sensitivity	Specificity	Positive predictive value	Negative predictive value	Positive likelihood ratio	Negative likelihood ratio	Accuracy
Lymphocyte count	0.331	0.012	0.75	50% (28.2-71.8)	28.9% (21.2-37.6)	10.8% (7.3-15.7)	77.1% (67-84.7)	0.7 (0.5-1.1)	1.73 (1.1-2.9)	32% (24.6-40.1)
Neutrophil to lymphocyte ratio	0.499	0.987	10.85	50% (28.2-71.8)	50.8% (41.8-59.7)	14.9% (10-21.6)	85.5% (79-90.1)	1.02 (0.7-1.6)	0.98 (0.6-1.6)	50.7% (42.4-58.9)
Platelet to lymphocyte ratio	0.702	0.003	311.18	68.2% (45.1-86.1)	68% (59.2-75.9)	26.8% (20-34.9)	92.6% (87-95.9)	2.1 (1.5-3.1)	0.5 (0.3-0.9)	68% (59.9-75.4)

AUC: area under the curve.

## Data Availability

The data used to support the findings of this study are included within the article.
